# Inhibition of IL-1β and TNF-α Secretion from Resting and Activated Human Immunocytes by the Homeopathic Medication Traumeel^®^ S

**DOI:** 10.1080/10446670410001722203

**Published:** 2004-06

**Authors:** Svetlana Porozov, Liora Cahalon, Michael Weiser, David Branski, Ofer Lider, Menachem Oberbaum

**Affiliations:** 1 The Center for Integrative Complementary Medicine Shaare Zedek Medical Center Jerusalem Israel; 2 The Department of Immunology The Weizmann Institute of Science Rehovot Israel; 3 Institute for Antiomotoxic Medicine and Matrix Regulation Research Baden-Baden Germany; 4 Department of Pediatrics Hadassah Medical Center Jerusalem Israel

## Abstract

Abstract Traumeel^®^ S (Traumeel), a mixture of highly diluted (10^-1^-10^-9^) extracts from medicinal plants and minerals is widely used in humans to relieve trauma, inflammation and degenerative processes. However, little is known about its possible effects on the behavior of immune cells. The effects of Traumeel were examined *in vitro* on the ability of resting and PHA-, PMA- or TNF-α-activated human T cells, monocytes, and gut epithelial cells to secrete the prototypic pro-inflammatory mediators IL-1β, TNF-α and IL-8 over a period of 24-72 h. Traumeel inhibited the secretion of all three agents in resting, as well as activated immune cells. IL-β secretion was reduced by up to 70% in both resting and activated cells; TNF-α secretion was reduced by up to 65 and 54%, respectively, and IL-8 secretion was reduced by 50% in both resting and activated cells (P<0.01 for all cells). Interestingly, the effect appeared to be inversely dose-related; maximal inhibition (usually 30-60% inhibition; P<0.01) was seen with dilutions of 10^-3^-10^-6^ of the Traumeel stock material. This finding suggests that Traumeel does not inhibit immune cells functions by exerting a toxic effect. Indeed, Traumeel did not affect T cell and monocyte proliferation. Although additional studies are needed to clarify the mode of action of Traumeel and to demonstrate causative relationship between the inhibition of cytokine/chemokine secretion in cell culture and the reported clinical effects of the preparation, our *in vitro* results offer a mechanism for the anti-inflammatory effects of Traumeel observed in clinical use.


*Clinical & Developmental Immunology*, June 2004, Vol. 11 (2), pp. 143–149

Taylor & Francis healthsciences

### Inhibition of IL-1β and TNF-α Secretion from Resting and Activated Human Immunocytes by the Homeopathic Medication Traumeel® S

SVETLANA POROZOV[a], LIORA CAHALON[b], MICHAEL WEISER[c], DAVID BRANSKI[d], OFER LIDER[b] and MENACHEM OBERBAUM[a,*]

[a]*The Center for Integrative Complementary Medicine, Shaare Zedek Medical Center, Jerusalem 91031, Israel;* [b]*The Department of Immunology, The Weizmann Institute of Science, Rehovot 76100, Israel;* [c]*Institute for Antiomotoxic Medicine and Matrix Regulation Research, Bahnackerstr 16, 76532 Baden-Baden, Germany;* [d]*Department of Pediatrics, Hadassah Medical Center, Jerusalem 91120, Israel*

Traumeel® S (Traumeel), a mixture of highly diluted ($10^{-1}$–$10^{-9}$) extracts from medicinal plants and minerals is widely used in humans to relieve trauma, inflammation and degenerative processes. However, little is known about its possible effects on the behavior of immune cells. The effects of Traumeel were examined *in vitro* on the ability of resting and PHA-, PMA- or TNF-α-activated human T cells, monocytes, and gut epithelial cells to secrete the prototypic pro-inflammatory mediators IL-1β, TNF-α and IL-8 over a period of 24–72 h. Traumeel inhibited the secretion of all three agents in resting, as well as activated immune cells. IL-β secretion was reduced by up to 70% in both resting and activated cells; TNF-α secretion was reduced by up to 65 and 54%, respectively, and IL-8 secretion was reduced by 50% in both resting and activated cells ($P < 0.01$ for all cells). Interestingly, the effect appeared to be inversely dose-related; maximal inhibition (usually 30–60% inhibition; $P < 0.01$) was seen with dilutions of $10^{-3}$–$10^{-6}$ of the Traumeel stock material. This finding suggests that Traumeel does not inhibit immune cells functions by exerting a toxic effect. Indeed, Traumeel did not affect T cell and monocyte proliferation. Although additional studies are needed to clarify the mode of action of Traumeel and to demonstrate causative relationship between the inhibition of cytokine/chemokine secretion in cell culture and the reported clinical effects of the preparation, our *in vitro* results offer a mechanism for the anti-inflammatory effects of Traumeel observed in clinical use.

*Keywords*: Cytokine secretion; Homeopathy; Inflammatory disorders; Trauma

### INTRODUCTION

Traumeel® S is a widely used medication containing a mixture of highly diluted ($10^{-1}$–$10^{-9}$ from mother solution) extracts of medicinal plants and minerals. It has been found beneficial to humans suffering from a wide spectrum of pathological conditions, including trauma, inflammation and degenerative processes (Zenner and Metelmann, 1992; Zenner and Weiser, 1997; Arora *et al.*, 2000; Oberbaum *et al.*, 2001). However, Traumeel includes only minute doses of its medicinal components. For example, the concentration of Mercury in Traumeel is lower than that allowed in drinking water in Germany. Such effects have made Traumeel one of the most popular alternative medications in Germany, where it is also used by conventional physicians (primarily orthopedists). A recent study showed the beneficial effect of Traumeel on chemotherapy-induced stomatitis in children undergoing bone marrow transplantations (Oberbaum *et al.*, 2001). Chemotherapy-induced stomatitis is one of the most severe side effects of chemotherapy; it has no effective treatment and often limits the intensity of chemotherapy (Wilkes, 1998).

However, despite the long use, popularity (Traumeel is one of the most popular alternative medicines in Germany, used also by conventional physicians) and good efficacy of Traumeel in a wide range of indications, its mode of action has been insufficiently studied. The current work analyzed the effects of Traumeel on human leukocyte function *in vitro*. Specifically, the effects of Traumeel were studied on T cell activation and on T cell, monocyte, endothelial cells and gut epithelial cell secretion of the major pro-inflammatory cytokines IL-1β, TNF-α and IL-8 (Feldman *et al.*, 2001; Apte and Voronov, 2002; Strieter, 2002). IL-1β was chosen, as it is a versatile and pivotal pro-inflammatory mediator. TNF-α is involved, along with IL-1β, in various aspects and reactions of the immune system, as well as in autoimmune and acute inflammatory

*Corresponding author. Tel.: +972-2-6666-395. Fax: +972-2-6666-975. E-mail: oberbaum@szmc.org.il; oberbaum@netvision.net.il

ISSN 1740-2522 print/ISSN 1740-2530 online © 2004 Taylor & Francis Ltd
DOI: 10.1080/10446670410001722203

diseases (Wilkes, 1998; Feldman *et al.*, 2001). IL-8 is a chemokine involved in the activation and recruitment of leukocytes, predominantly neutrophils, from blood vessels to extravascular sites of inflammation.

### MATERIAL AND METHODS

### Materials

Traumeel was obtained, free of charge, from Biologische Heilmittel Heel GmbH (Baden-Baden, Germany). Traumeel® S is supplied in glass ampoules prepared for injection, each containing 2.2 ml of the medication. The active ingredients of Traumeel are prepared in accordance with the German Homeopathic Pharmacopoeia. The following reagents and chemicals were used: human recombinant TNF-α, phorbol myristate acetate (PMA), BSA, heparin (Sigma; St. Louis, MO, USA), RPMI 1640 medium (Gibco BRL), M199 medium, HEPES buffer, antibiotics, heat-inactivated fetal calf serum (FCS), glutamine, sodium pyruvate (Kibbutz Beit-Haemek, Israel).

### Cells and Cell Culture

T cells from healthy human peripheral blood (PBL) were isolated on Ficoll gradients, washed, resuspended in PBS containing 3% heat-inactivated FCS, and incubated (45 min, 37°C, 7% $CO_2$-humidified atmosphere) on nylon–wool columns (NovaMed; Jerusalem, Israel), as described (Franitza *et al.*, 2000; Ariel *et al.*, 2002). Non-adherent cells were eluted and washed, and platelets were removed by centrifugation (700 rpm, 15 min, 18°C). Residual monocytes were removed by incubating (2 h, 37°C) the cells on tissue culture plates, and collecting the non-adherent cells. The PBL thus obtained contained $>95\%$ $CD3^+$ cells, as determined by FACScan analysis.

Where indicated, an *in vitro* co-culture system was used involving two human cell lines—Jurkat (T cells) and the monocytic cell line THP-1. PBLs, Jurkat T cells and THP-1 monocytes were cultured in RPMI 1640 medium (10% heat-inactivated FCS, 1% antibiotics, 1% glutamine and 1% pyruvate) in 75-$cm^2$ tissue culture flasks (Falcon) singly.

Freshly isolated human umbilical vein endothelial cells (HUVEC) were cultured in M199 medium containing 20% FCS, 1% glutamine, 0.1% gentamicin, 1% HEPES, 0.1% heparin and 0.1% EGF. Human colonic epithelial line cells, designated HT-29, were cultured in McCoy's medium, supplemented with 10% heat-inactivated FCS, 1% glutamine and 1% antibiotics, as previously described (Chowers *et al.*, 2001). HUVEC and HT-29 cells were grown in 24-well tissue culture plates (Corning Inc., Corning, NY).

### Determination of IL-1β, TNF-α and IL-8 by ELISA

To measure cytokine secretion, HT-29 and HUVEC cells were seeded onto 24-well plates and grown until confluent monolayers were formed. To determine the IL-1β and IL-8 secretions, the indicated cells were analyzed in two different series of experiments: (1) cells were incubated in fresh medium containing various dilutions of Traumeel (usually $10^{-1}$–$10^{-7}$) for 24, 48 or 72 h; (2) the cells were treated with Traumeel (24 h) before TNF-α-activation (100 ng/ml; 24 h). Alternatively, the cells were incubated with their respective activator before exposure to Traumeel. PBLs were seeded onto the 24-well plates ($2 \times 10^6$ cells/ml/well) and stimulated with PHA (1 μg/ml; 24 h) under the same conditions described previously. To measure cytokine secretion by the two cell lines, the Jurkat and THP-1 cells were mixed ($2 \times 10^6$ and $2.5 \times 10^5$ cells/ml, respectively; 1 ml/well), and treated for 48 h with PHA (4 μg/ml) and PMA (50 ng/ml) before exposing the cells to Traumeel (24 h). In a separate series of experiments, the cells were preincubated with Traumeel (24 h) and subsequently treated with the activators. Control cells were incubated only with Traumeel (24 or 72 h). Following the different treatments, the supernatants were collected, cleared by centrifugation and stored at $-20$°C until evaluation by ELISA. All cytokine measurements were carried out using conventional ELISA kits and reagents (BioSource International, Inc., USA), according to the manufacturer's instructions (Chowers *et al.*, 2001).

### Statistical Analysis

The results shown are from one representative experiment out of a minimum of three identical experiments that yielded comparable results or, when indicated, the average of identical experiments. The data are expressed as means ($\pm$ SD) of cytokine/chemokine content in supernatants of triplicate or quadruplicate wells. Statistical analysis of the differences between the means of the different groups within a given experiment was evaluated using the paired Student's *t*-test.

### RESULTS

### Effects of Traumeel on IL-1β Secretion from Resting, Co-cultured Jurkat and THP-1 Cells

Different concentrations of Traumeel were added to co-cultures of Jurkat and THP-1 cells which were maintained under tissue culture conditions for either 24 or 72 h. IL-β secretion was measured by ELISA. Note as shown in Fig. 1 and in additional figures in which the secretion of other cytokines was tested, that the spontaneous release of TNF-α is indicated as the secreted protein in the absence of Traumeel. The results, shown in Fig. 1A, indicate that while there was no effect on IL-1β secretion when cells were incubated with Traumeel for 24 h, when the co-cultured cells were treated with Traumeel for 72 h, a dose-dependent effect was obtained. Interestingly, Traumeel in a dilution of 1:10 markedly

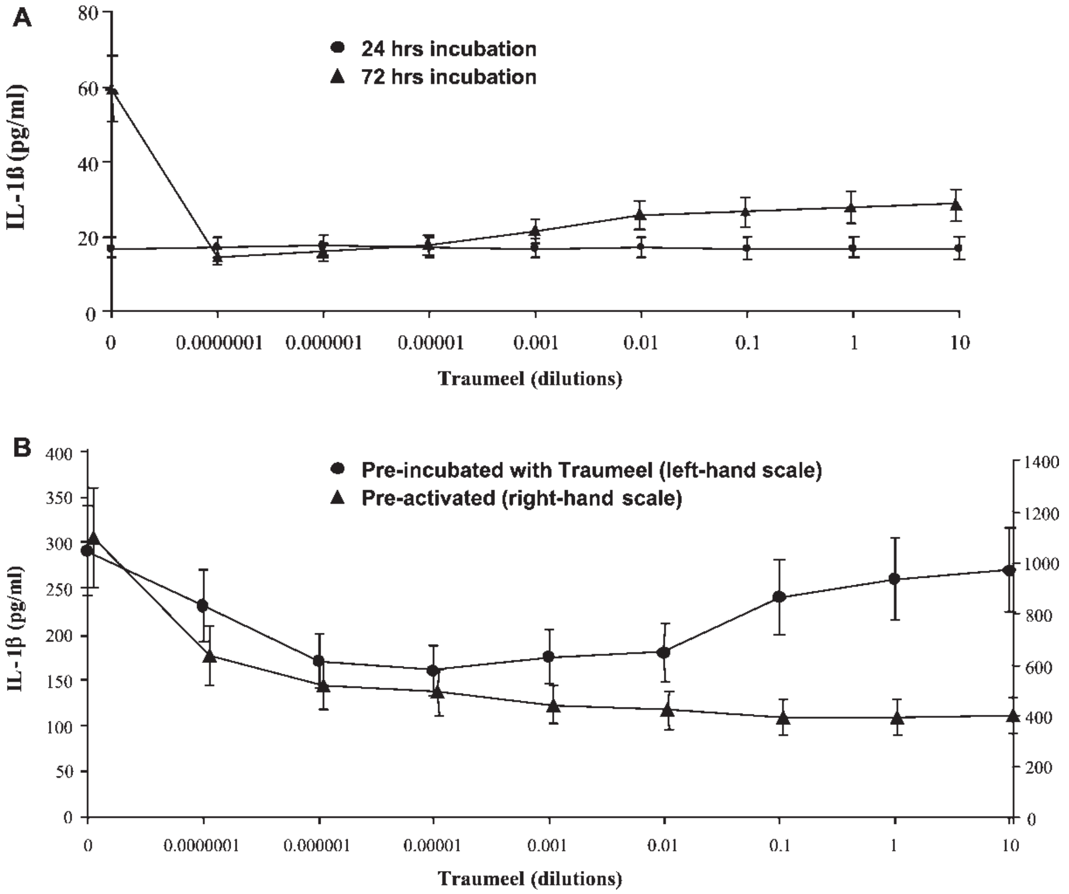

FIGURE 1 Effects of Traumeel on IL-1β secretion from interacting, resting and PHA- and PMA-activated Jurkat and THP-1 cells. (A) The human $CD4^+$ line of T cells and monocytes (THP-1) were co-cultured under tissue culture conditions and incubated with the indicated concentrations (serial dilutions) of Traumeel for 24 h (circles) and 72 h (triangles). Each data point represents the mean (± SD) of triplicate wells. One experiment representative of five. (B) Human T cell lines were either pre-exposed to Traumeel and subsequently activated with PHA and PMA for 48 h (circles) or activated with PHA and PMA for 48 h, washed and exposed to Traumeel for an additional period of 24 h (triangles). Each data point represents the mean (± SD) of triplicate wells. One experiment representative of three.

inhibited IL-1β secretion from the co-cultured resting T cells and monocytes (from 60 to 24 pg/ml; $P < 0.05$); further dilutions of up to $10^{-7}$ exerted an inversely dose–response inhibition of the basal levels of IL-1β secretion, from 24 to 18 pg/ml, at dilutions of $10^{-1}$–$10^{-7}$, respectively.

Further, cells of both types were exposed for 24 h to different dilutions of Traumeel, either before or after their activation with PHA and PMA (for 48 h). The results indicate that Traumeel inhibited IL-1β secretion when used either before or after PHA activation (Fig. 1B). However, whereas pretreatment of cells with Traumeel led to an inverse bell-shaped dose–response curve reaching the maximal inhibition at $10^{-5}$ dilution, late exposure of the activated cells to Traumeel yielded a reversed dose–response curve, reaching a maximal inhibition of IL-1β secretion at $10^{-2}$ dilution (from 1100 pg/ml at baseline to 330 pg/ml). These pattern of dose-dependency effects of Traumeel also implies that this medication do not affect T cell function via cytotoxicity. In fact, in a separate set of studies, we could not detect any effect on T cell viability and proliferation, even when the cells were exposed to Traumeel for 3 days (not shown).

### Effects of Traumeel on TNF-α Secretion from Jurkat and THP-1 Cells

Non-activated immunocytes were incubated with different concentrations of Traumeel for 24 and 72 h. TNF-α secretion was measured in the supernatants at the end of the assay, using ELISA. The results, shown in Fig. 2A, indicate that Traumeel significantly inhibits TNF-α secretion ($P < 0.01$) when diluted to $10^{-3}$–$10^{-7}$. Similar to the effects on IL-1β, inhibition was more evident after prolonged Traumeel incubation. Moreover, an inverse dose–response pattern of inhibition of TNF-α secretion was observed when the cells were incubated with Traumeel for 72 h (Fig. 2A).

The modulatory effects of Traumeel on PHA and PMA-activated Jurkat and THP-1 cells were evaluated as described in the legend to Fig. 1. The results, shown in Fig. 2B, demonstrate a similar inhibition pattern of TNF-α secretion by Traumeel, irrespective of whether the cells

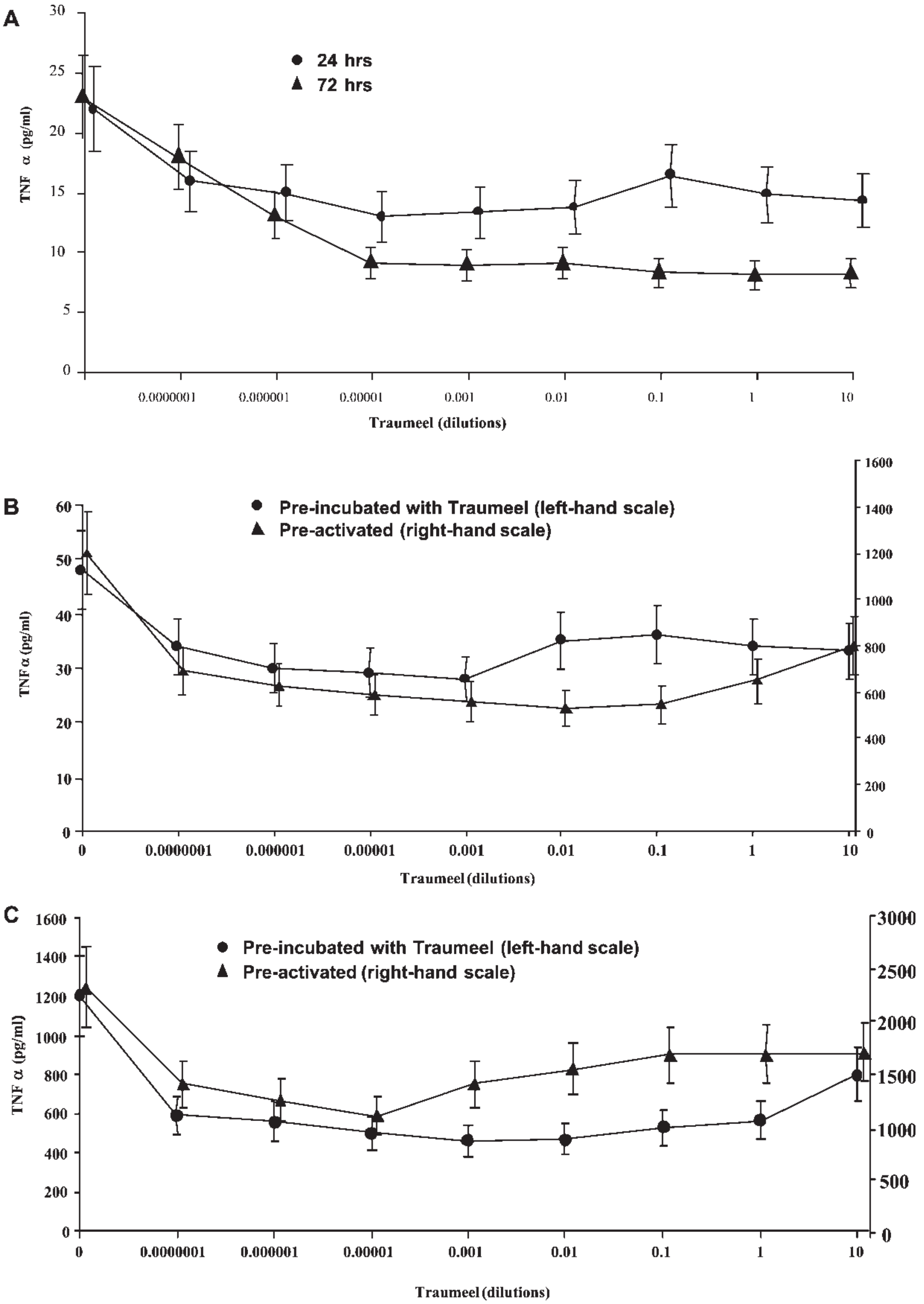

FIGURE 2 Effects of Traumeel on TNF-α secretion from interacting, resting and PHA- and PMA-activated Jurkat and THP-1 cells (A, B) and from activated PBL cells (C). (A) THP-1 and Jurkat cells were maintained under tissue culture conditions while exposed to the indicated serial dilutions of Traumeel for 24 h (circles) and 72 h (triangles). Each data point represents the mean (± SD) of triplicate ELISA wells. One experiment representative of four. (B) Human cells were either pre-exposed to Traumeel and subsequently activated with PHA and PMA for 48 h (circles) or activated with PHA and PMA for 48 h, washed and exposed to Traumeel for an additional period of 48 h (triangles). Each data point represents the mean (± SD) of triplicate wells. One experiment representative of five. (C) Human PBL cells were either pre-exposed to Traumeel for 24 h and subsequently activated with PHA for 24 h (circles) or activated with PHA for 24 h, washed and exposed to Traumeel for an additional period of 24 h (triangles). Each data point represents the mean (± SD) of triplicate wells. One experiment representative of four.

were exposed to Traumeel prior to or after the two activators. Basal levels of TNF-α secretion were significantly lower ($P < 0.05$) when cells were exposed to Traumeel before activation. Under both conditions, the inhibitory effect of Traumeel on TNF-α secretion was evident at concentrations of $10^{-1}$–$10^{-4}$ ($P < 0.01$). However, at the higher dilutions of Traumeel, the level of inhibition of TNF-α secretion gradually decreased until it reached basal secretion levels.

### Effects of Traumeel on TNF-α Secretion from Freshly Isolated Human T Cells

The previous experiments indicate that Traumeel can affect the secretion of IL-1β and TNF-α from Jurkat and THP-1 human T cell lines. To examine whether Traumeel can modulate TNF-α secretion from T cells isolated from healthy human donors, such T cells were treated with Traumeel (24 h) shortly after their separation and subsequently activated with PHA (24 h), or vice versa. The level of secreted TNF-α was measured by ELISA. The results, shown in Fig. 2C, indicate an inhibition at $10^{-1}$–$10^{-4}$ dilutions of Traumeel ($P < 0.05$). Similarly to the effects observed with activated human T cell lines, there was no inhibition at dilutions of $10^{-5}$–$10^{-7}$.

### Effects of Traumeel on IL-8 Secretion from Non-activated and Activated HT-29 Cells

The effects of Traumeel on the secretion of IL-8 from the monocytic cell line THP-1 was also investigated. Figure 3A shows a reverse dose–response inhibition by Traumeel, with a maximal inhibitory effect of 40–50% ($P < 0.05$) occurring at $10^{-4}$–$10^{-5}$ dilutions.

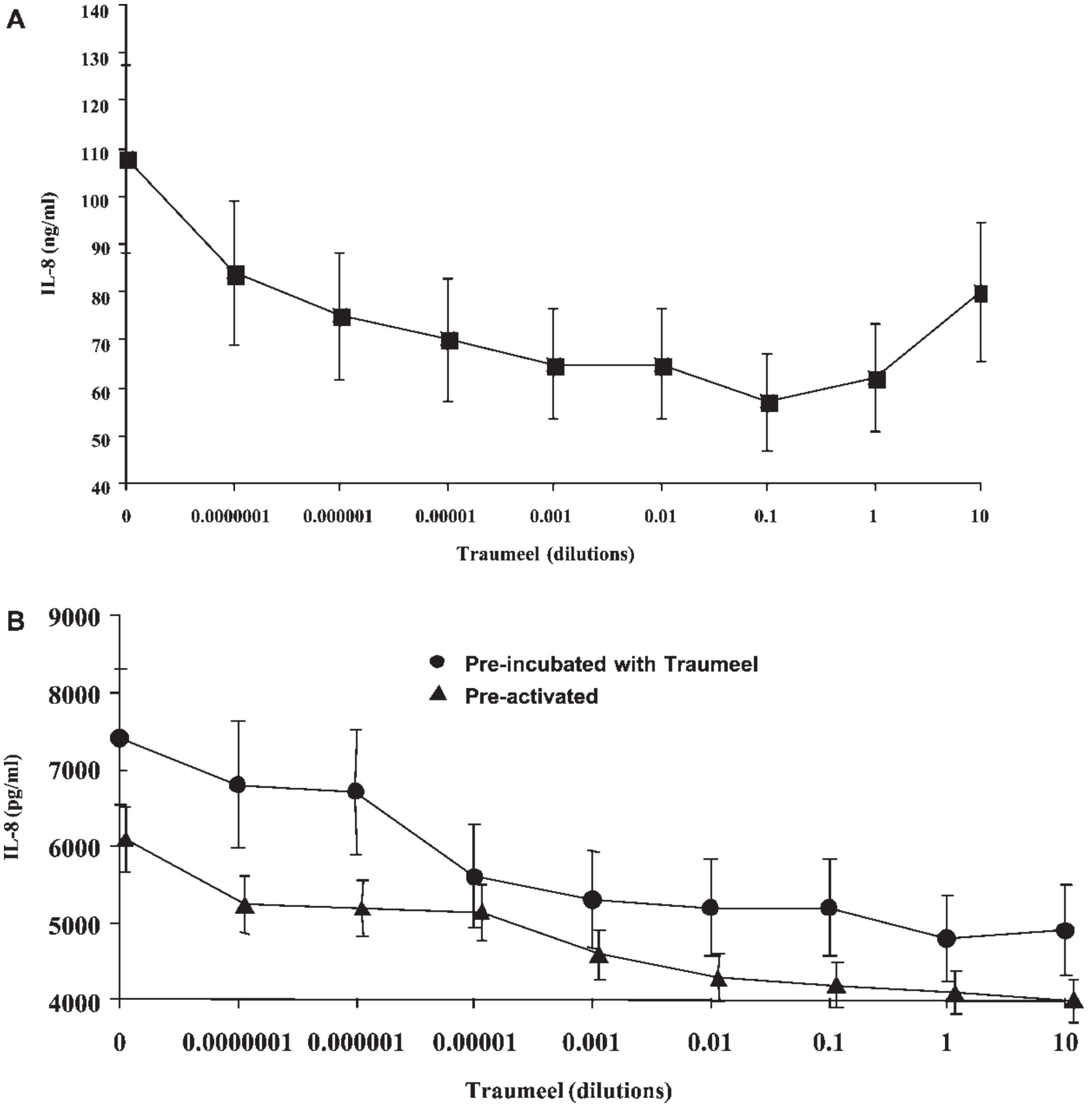

FIGURE 3 Effects of Traumeel on IL-8 secretion from resting (A) and activated (B) HT-29 cells. (A) Cells from the human gut epithelial cell line, HT-29, were incubated (24 h) with the indicated concentrations of Traumeel and Il-8 secretion was measured using ELISA. Each data point represents the mean (± SD) of triplicate wells. One experiment representative of three. (B) Cells were pre-incubated with Traumeel, and subsequently activated with TNF-α for 24 h (circles), or activated with TNF-α for 24 h, and subsequently exposed to the indicated concentrations of Traumeel for 24 h (triangles). Each data point represents the mean (± SD) of triplicate wells. One experiment representative of four.

Further, the effects of Traumeel on IL-8 secretion from TNF-α-activated THP-1 cells were studied. The results, shown in Fig. 3B, indicate that irrespective of whether the cells were first exposed to Traumeel and subsequently activated or vice versa, there was a gradual inhibition of IL-8 secretion, reaching a maximal effect of 40–50% inhibition ($P < 0.05$) at dilutions of $10^{-5}$–$10^{-7}$.

### Effects of Traumeel on IL-1β Secretion from Human Gut Epithelial Cells

The effects on IL-1β secretion by the human gut epithelial cell line HT-29 by different dilutions of Traumeel (24 and 48 h) were investigated. The results, shown in Fig. 4A, indicate an inhibition of IL-1β secretion of 30–50% when cells were exposed to Traumeel for 24 or 48 h. Unlike the gradual and consistent effect at low dilution ($10^{-4}$–$10^{-7}$) on mobile immunocytes, inhibition of IL-1β secretion from the non-activated epithelial cells was reduced at higher dilutions.

Finally, the putative effect of Traumeel on the secretion of IL-1β from TNF-α-activated HT-29 cells was examined, assuming that such an activation may occur *in vivo* during inflammation. At 1–10% dilutions, there was significant inhibition of IL-1β secretion (80%; $P < 0.01$; Fig. 4B).

### DISCUSSION

Traumeel has been on the market for approximately 80 years, and has a long record of use in millions of patients (Zenner and Weiser, 1997; Arora *et al.*, 2000). It has been postulated that Traumeel has beneficial anti-traumatic and anti-inflammatory activities as indicated in a wide range of cases (Lussignoli *et al.*, 1999; Oberbaum *et al.*, 2001). However, in contrast to its wide clinical use, little is known as to whether (and how) Traumeel affects immune cell functions related to inflammation. This study reports that the homeopathic remedy Traumeel, at dilutions of $10^{-1}$–$10^{-7}$, inhibits, in a unique dose-dependent fashion, the secretion of the pro-inflammatory cytokines IL-1β TNF-α and the chemokine IL-8, from (mobile) human leukocytes and (resident) gut epithelial cells *in vitro*. Interestingly, Traumeel appears to negatively affect the secretion of the tested cytokine and chemokine, from either

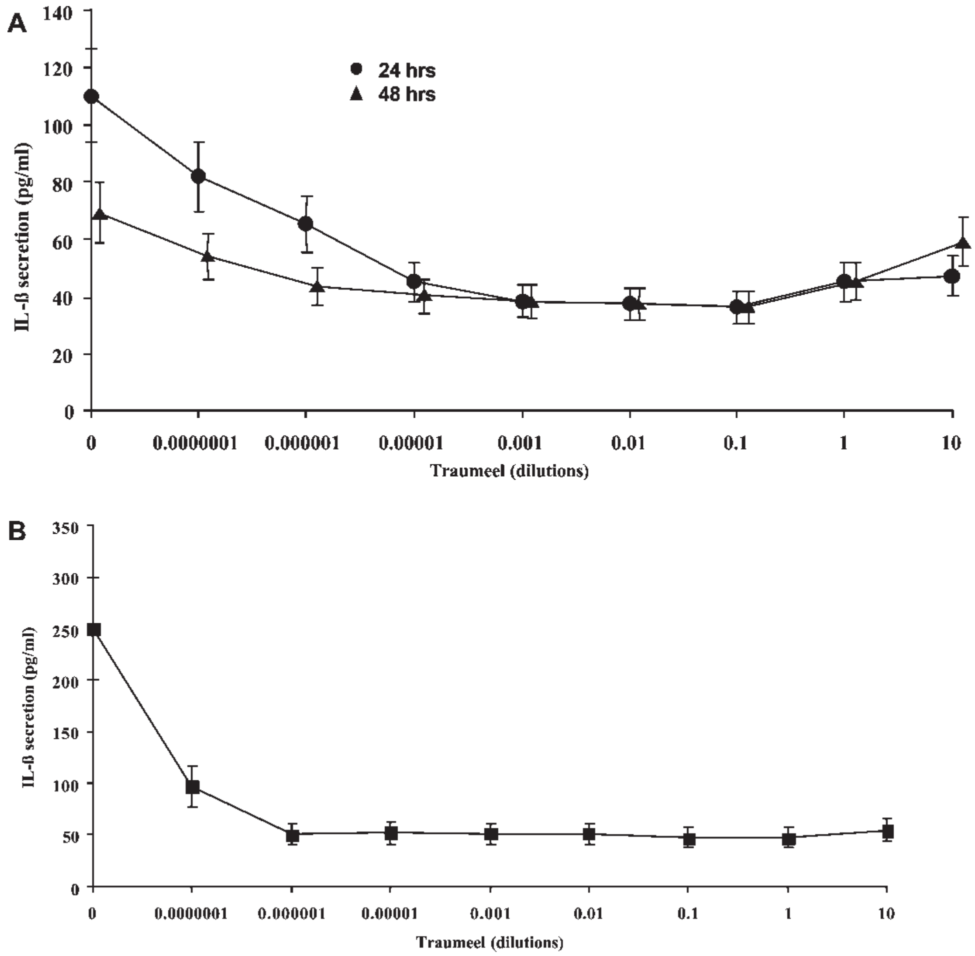

FIGURE 4 Effects of Traumeel on IL-1β secretion from resting (A) and TNF-α-activated (B) HT-29 cells. (A) HT-29 cells were exposed to Traumeel for 24 h (circles) or 48 h (triangles) prior to the measurement of IL-1β secretion. Each data point represents a mean (± SD) of triplicate wells. One experiment representative of four. (B) Cells were activated with TNF-α (24 h), and subsequently exposed to the indicated amounts of Traumeel. Each data point represents the mean (± SD) of triplicate wells. One experiment representative of five.

the mobile leukocytes or resident gut epithelial cells, in an inversely dose–response pattern. This phenomenon was observed in all the tested cell types or their combinations, regardless of whether the cells were non-activated or activated with PHA/PMA or TNF-α. In none of the experiments did Traumeel increase the secretion of the pro-inflammatory mediators, whether or not the cells were activated by other means, implying that the medication lacks any activating (or inflammatory) capacity. The results support the characterization of Traumeel as an anti-inflammatory medication.

The human cell types studied were chosen because they represent either the mobile arm of the immune system in the form of blood-borne T cells (freshly isolated or of the Jurkat $CD4^+$ T cell line) and monocytes (THP-1 cell line), or the first line of immune defense of the gut-associated immune system represented by the resident, non-mobile gut epithelial cells (HT-29 cell line). In all cases, Traumeel exerted comparable inhibitory effects on the secretion of the pro-inflammatory mediators. It is noteworthy that the apparent inhibitory concentrations of Traumeel, i.e. 10-3-10-8, are similar to those active *in vivo* in stomatitis (Oberbaum *et al.*, 2001).

There is a great need for rigorous studies of the actions of remedies such as Traumeel, which are frequently used in the practice of complementary medicine and homeopathy (Jonas *et al.*, 2003). The current investigation shows that Traumeel treatment influences immune cell functions related to inflammation *in vitro*. Inhibition of IL-1β, TNF-α and IL-8 secretion from resting or (PHA-, PMA-, or TNF-α)-activated immunocytes by Traumeel was clearly shown. However, the results presented leave many questions unanswered, e.g. the mode of action, and whether Traumeel affects other facets of immune cell behavior during inflammation such as proliferation, expression of activation-related receptors, adhesion to other cells or to components of blood vessel walls and extracellular matrix. It is conceivable but unproven that Traumeel interferes with specific intracellular signal transduction pathways. Furthermore, Traumeel is a mixture of several plant extracts and minerals and the contribution of each ingredient, as well as possible synergistic effects of the composition, needs further study.

As to the inversely dose-dependent effects, we can only speculate that the optimal immuno-modulatory effect of the mixture requires exact concentrations of the active compounds. Hence, Traumeel at too high or too low dilutions would fail to exert an inhibitory effect on cytokine secretion. Be that as it may, it was noteworthy that Traumeel did not show any activating effects even at the higher concentrations $10^{-1}–10^{-3}$. Be that as it may, these results also imply that the above-mentioned effects of Traummel are not due to toxic effect of the medication. Indeed, in a separate set of studies, we could not detect any effect of Traumeel on T cell and monocyte proliferation (and viability), even if the cells were exposed to the active concentration of the medication for 72 h (not shown). Interestingly, we had previously found that a naturally-occurring breakdown product of heparan sulfate proteoglycan, which is generated *in vivo* by the action of heparanase, also inhibited TNF-α secretion, but not T-cell proliferation (Lider *et al.*, 1995).

The mode of interaction with immune cells also would need detailed investigation. It remains to be established whether Traumeel interacts with specific cell surface receptors or penetrates the cell membranes. However, all such theories, although testable, remain speculation at present.

The current results should challenge researchers to study the indicated anti-activating and anti-inflammatory potential of Traumeel, both *in vitro* and *in vivo*, in animal models of inflammation. If such studies corroborate the findings presented in the current work, this would be strong support for the use of Traumeel and possibly of related medications as conventional therapeutic modalities administered to patients suffering from chronic or acute inflammatory isorders.

### *Acknowledgements*

This research was supported by the Center for Absorption in Science, Ministry of Immigrant Absorption, State of Israel.

### *References*

Apte, R.N. and Voronov, E. (2002) "IL-1—a major pleiotropic cytokine in tumor host interactions", *Semin. Cancer Biol.* **12**, 277–290.
Ariel, A., Novick, D., Rubinstein, M., Dinarello, C.A., Lider, O. and Hershkoviz, R. (2002) "IL-12 and IL-18 induce MAP kinase-dependent adhesion of T cells to extracellular matrix components", *J. Leuk. Biol.* **72**, 192–198.
Arora, S., Harris, T. and Scherer, C. (2000) "Clinical safety of a homeopathic preparation", *Biomed. Ther.* **18**, 222–225.
Chowers, Y., Lider, O., Schor, H., *et al.* (2001) "Disaccharides derived from heparin or heparan sulfate regulate IL-8 and IL-1β secretion by intestinal epithelial cells", *Gastroenterology* **120**, 449–459.
Feldman, M., Brennan, F.M., Foxwell, B.M. and Maini, R.N. (2001) "The role of TNF-α and IL-1 in rheumatoid arthritis", *Curr. Dir. Autoimmun.* **3**, 188–199.
Franitza, S., Hershkoviz, R., Kam, N., *et al.* (2000) "TNF-α associated with extracellular matrix fibronectin provides a stop signal for chemotactically migrating T cells", *J. Immunol.* **165**, 2738–2747.
Jonas, W.B., Kaptchuk, T.J. and Linde, K. (2003) "A critical overview of homeopathy", *Ann. Int. Med.* **138**, 393–400.
Lider, O., Cahalon, L., Gilat, D., *et al.* (1995) "A disaccharide that inhibits tumor necrosis factor-α is formed from the extracellular matrix by the enzyme heparanase", *Proc. Natl Acad. Sci. USA* **92**, 5037–5041.
Lussignoli, S., Bertani, S., Metelmann, H., Bellavite, P. and Conforti, A. (1999) "Effects of Traumeel S: a homeopathic formulation, on blood-induced inflammation in rats", *Comp. Ther. Med.* **7**, 225–230.
Oberbaum, M., Yaniv, I., Ben-Gl, Y., *et al.* (2001) "A randomized, controlled clinical trial of the homeopathic medication Traumeel S in the treatment of chemotherapy-induced stomatitis in children undergoing stem cell transplantation", *Cancer* **92**, 684–690.
Strieter, R.M. (2002) "IL-8: a very important chemokine of the human airway epithelium", *Am. J. Physiol. Lung Cell Mol. Physiol.* **283**, 690–699.
Wilkes, R.W. (1998) "Prevention and treatment of oral mucositis following cancer chemotherapy", *Semin. Oncol.* **25**, 538–551.
Zenner, S. and Metelmann, H. (1992) "Application possibilities of Traumeel S injection solution: results of a multicentric monitoring trial conducted in 3241 patients", *Biol. Ther.* **10**, 301–310.
Zenner, S. and Weiser, M. (1997) "Oral treatment of traumatic, inflammatory, and degenerative conditions with a homeopathic remedy", *Biomed. Ther.* **15**, 22–26.

